# Association of Thyroid Autoimmunity With Age-Related Macular Degeneration in U.S. Adults

**DOI:** 10.1167/tvst.15.11.3

**Published:** 2026-11-02

**Authors:** Xiaodong Chen, Jia Zhang, Tien Yin Wong, Lvzhen Huang, Ya Xing Wang

**Affiliations:** 1 Beijing Visual Science and Translational Eye Research Institute (BERI), Beijing Tsinghua Changgung Hospital, Tsinghua Medicine, Tsinghua University, Beijing, China; 2 Department of Ophthalmology, Peking University People's Hospital, Beijing, China; 3 Beijing Key Laboratory of Ocular Disease and Optometry Science, Peking University People's Hospital, Beijing, China

**Keywords:** age-related macular degeneration, thyroid autoimmunity, thyroglobulin antibodies, thyroid peroxidase antibodies, NHANES, Mendelian randomization

## Abstract

**Purpose:**

To investigate the associations of thyroid autoimmunity, assessed by thyroid peroxidase antibody (TPOAb) and thyroglobulin antibody (TgAb), with prevalent age-related macular degeneration (AMD) in U.S. adults, and to explore whether genetic liabilities to Hashimoto thyroiditis (HT) and Graves’ disease (GD) were associated with AMD risk using two-sample Mendelian randomization (MR).

**Methods:**

This cross-sectional study included 2967 adults aged 40 years or older from the 2007–2008 National Health and Nutrition Examination Survey. Survey-weighted logistic regression models included TPOAb and TgAb positivity simultaneously. Two-sample MR analyses were exploratory.

**Results:**

Among 2967 participants, 234 had AMD, corresponding to a weighted prevalence of 6.6% (95% confidence interval [CI], 5.6%–7.9%). In the mutually adjusted observational model, TgAb positivity was associated with higher odds of prevalent AMD (odds ratio [OR], 2.02; 95% CI, 1.13–3.61; *P* = 0.022), whereas TPOAb positivity was not associated with AMD (OR, 0.93; 95% CI, 0.63–1.37; *P* = 0.676). Exploratory MR analyses suggested a possible association between genetic liability to HT and AMD (OR, 1.14; 95% CI, 1.01–1.29; *P* = 0.033), whereas evidence for GD was less consistent.

**Conclusions:**

TgAb positivity was associated with prevalent AMD. The cross-sectional design and the nonidentical exposures assessed in the observational and MR analyses preclude causal inference.

**Translational Relevance:**

Thyroglobulin antibody positivity was associated with prevalent AMD and may reflect a systemic immune phenotype warranting prospective investigation.

## Introduction

Age-related macular degeneration (AMD) is a leading cause of irreversible central vision loss in older adults.^1^ In 2020, an estimated 196 million individuals had AMD worldwide, and approximately 1.8 million adults aged 50 years or older were blind because of AMD.^2^^,^^3^ Although researchers have revealed many risk factors of AMD including aging,^4^ smoking,^5^ and cardiovascular or metabolic conditions,^6^ the biological mechanisms underlying disease onset and progression remain incompletely understood. Accumulating evidence suggests that chronic inflammation, immune dysregulation, and complement-related pathways play important roles in AMD pathobiology.^7^^–^^9^

Autoimmune thyroid disease, including Hashimoto thyroiditis (HT) and Graves’ disease (GD), is the most common organ-specific autoimmune disorder and is characterized by circulating thyroid autoantibodies, particularly thyroid peroxidase antibodies (TPOAb) and thyroglobulin antibodies (TgAb).^10^^,^^11^ A thyroid-retina link is biologically plausible because thyroid hormone signaling contributes to retinal development and survival, and thyroid dysfunction has broad systemic vascular and metabolic effects.^12^^,^^13^ Epidemiologic evidence also suggests that thyroid-related traits may be associated with AMD. Higher free thyroxine concentrations were associated with greater AMD risk in the Rotterdam Study.^14^ Overt hyperthyroidism was associated with 10-year incident AMD in the Blue Mountains Eye Study.^15^ In addition, a recent meta-analysis including 13 epidemiologic studies reported that thyroid disease was associated with a higher risk of AMD.^16^ Genetic evidence has been more specific, with Mendelian randomization (MR) analyses implicating higher genetically predicted free thyroxine in late AMD.^17^^,^^18^

However, prior studies have focused primarily on thyroid hormone status or clinically recognized thyroid disease rather than thyroid autoimmunity itself.^14^^,^^15^^,^^19^^,^^20^ This distinction may be important because TPOAb and TgAb reflect thyroid autoimmunity and may be detectable before overt thyroid dysfunction becomes clinically apparent.^10^^,^^21^ Given growing interest in systemic immune mechanisms in AMD, thyroid autoantibodies may provide information beyond thyroid hormone measurements alone.^7^^–^^9^^,^^21^

We therefore investigated the associations of TPOAb and TgAb with AMD in a population-based sample of U.S. adults from the National Health and Nutrition Examination Survey (NHANES) and additionally explored whether genetic liability to autoimmune thyroid disease was associated with AMD risk using two-sample MR.

## Methods

### Participants in NHANES

NHANES is a continuous, cross-sectional survey designed to assess the health and nutritional status of the noninstitutionalized U.S. population. We used data from the 2007–2008 cycle, which included 10,149 participants. We excluded 6124 participants aged younger than 40 years, 834 without gradable AMD information, and an additional 224 without the required TPOAb or TgAb measurements, leaving 2967 participants for analysis (Fig.).

**Figure.**
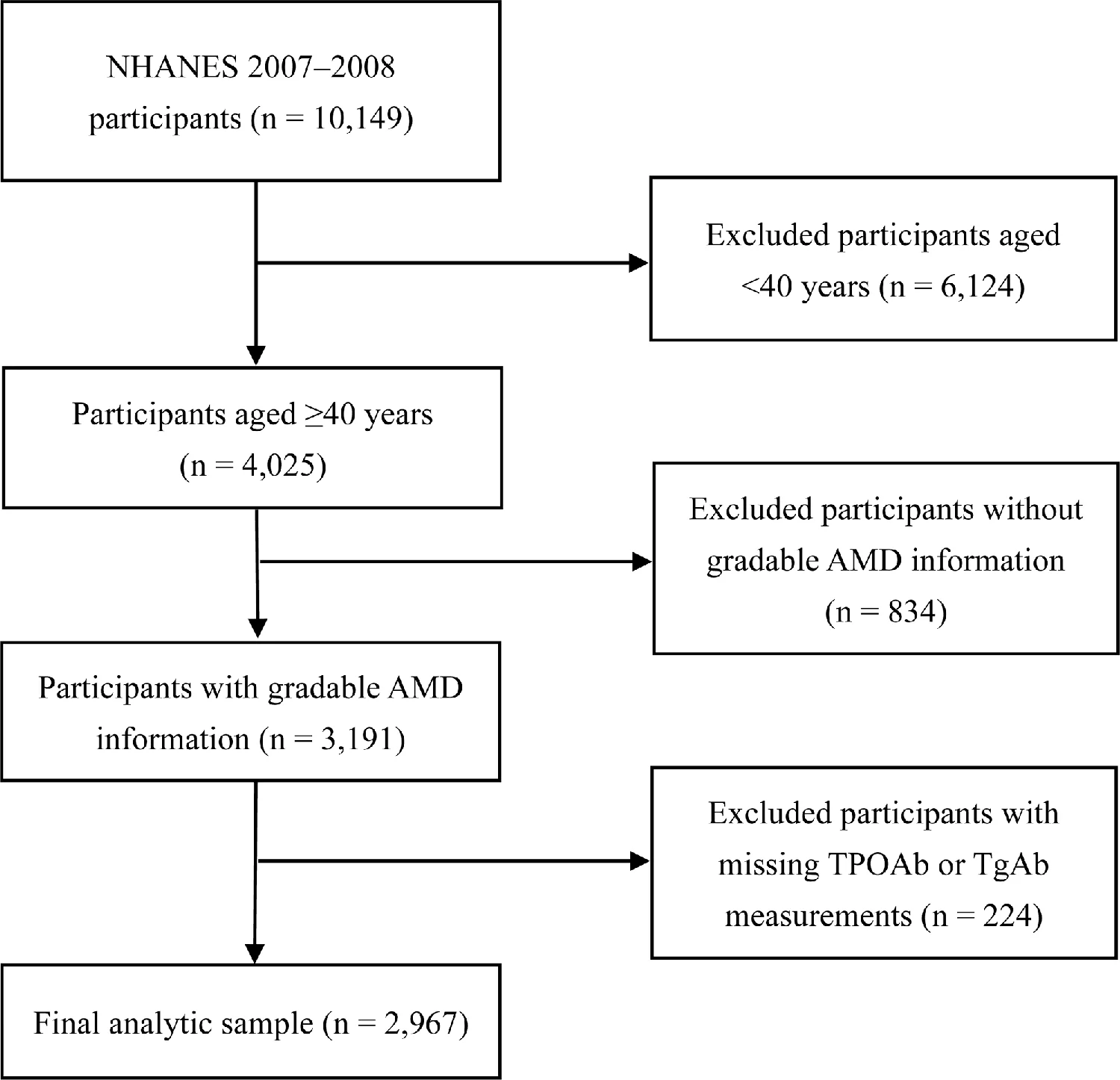
Selection of participants.

NHANES protocols were approved by the National Center for Health Statistics Research Ethics Review Board, and all participants provided written informed consent. The NHANES protocols were conducted in accordance with the Declaration of Helsinki. This secondary analysis used publicly available deidentified data. Because this study used publicly available deidentified data, additional institutional review board approval was not required.

### Exposure

The titers of TgAb and TPOAb were examined through a two-step immunoenzymatic assay. Based on prior studies,^22^^–^^24^ TPOAb and TgAb positivity were defined as concentrations of ≥9 IU/mL and ≥4 IU/mL, respectively. TgAb positivity was evaluated with TPOAb positivity included simultaneously in the mutually adjusted regression models.

Secondary analyses evaluated four mutually exclusive antibody profiles: neither antibody, TPOAb positivity only, TgAb positivity only, and positivity for both antibodies. Antibody concentrations were additionally evaluated as log2-transformed continuous variables, with odds ratios (ORs) representing the association per doubling of concentration. These analyses were considered exploratory.

### Retinal Photography and AMD Grading

Two 45° nonmydriatic retinal photographs were captured from each eye in the NHANES 2007-2008 cycle using the Canon CR6-45NM fundus imaging system (Canon Inc., Tokyo, Japan). The presence of AMD was assessed by two specialists.^25^^,^^26^ Participant-level AMD status was defined using OPDUARM, the NHANES three-level classification based on the worse-affected eye: no AMD, early AMD, or late AMD. Early AMD was defined according to the NHANES grading algorithm based on drusen characteristics and pigmentary abnormalities; late AMD comprised geographic atrophy or signs of neovascular AMD. For the primary outcome, early and late AMD were combined as any prevalent AMD.

### Covariates in NHANES

Covariates included age, sex, race/ethnicity (Mexican American, other Hispanic, non-Hispanic White, non-Hispanic Black, and other race), body mass index (BMI), smoking status, poverty-income ratio (PIR), education level, hypertension, diabetes, and cardiovascular disease. Cardiovascular disease was defined as a composite of self-reported congestive heart failure, coronary heart disease, angina, myocardial infarction, or stroke. Thyroid-stimulating hormone, free thyroxine, free triiodothyronine, and thyroid-related medication use were evaluated in sensitivity analyses.

### MR Analysis

A two-sample MR analysis was applied to investigate whether genetic liability to HT and GD was associated with AMD risk. Summary statistics for HT and GD were obtained from European-ancestry GWAS meta-analyses including UK Biobank and FinnGen,^27^ and summary statistics for AMD were obtained from FinnGen (Supplementary Table S1).^28^ Independent instrumental variables were selected using a genome-wide significance threshold of *P* < 5×10^−^^8^ and linkage disequilibrium clumping (*r*^2^ < 0.001, window size 10,000 kb). Instrument strength was assessed using the F statistic, calculated as the squared ratio of the SNP-exposure estimate to its standard error, and variants with F statistics ≤10 were excluded. Detailed information on the retained instruments is provided in Supplementary Table S2.

Exposure and outcome alleles were harmonized to the same effect allele. Palindromic variants with intermediate allele frequencies and ambiguous strand orientation were excluded. The inverse variance weighted (IVW) multiplicative random-effects model was used as the primary MR analysis, with fixed-effects IVW, MR-Egger, weighted median, and weighted mode analyses used as complementary methods. Heterogeneity and directional horizontal pleiotropy were assessed using Cochran's Q test and the MR-Egger intercept test, respectively. Leave-one-out analyses were performed by sequentially excluding each instrument, and Steiger directionality tests were conducted to assess whether the instruments explained more variance in the specified exposure than in the outcome.^29^ Because FinnGen contributed to both the thyroid-disease exposure GWAS meta-analyses and the AMD outcome GWAS, partial participant overlap was possible. Reverse MR analysis was additionally performed to examine the potential effect of AMD on HT and GD. All MR analyses were conducted in R version 4.3.2.

### Statistical Analysis

All survey-weighted analyses incorporated the 2-year Mobile Examination Center examination weight (WTMEC2YR), strata (SDMVSTRA), and primary sampling units (SDMVPSU) to account for the complex, multistage NHANES sampling design. Continuous variables were summarized as weighted means with weighted standard deviations, and categorical variables as unweighted counts with weighted percentages. Survey-weighted prevalence estimates and 95% confidence intervals (CI) were calculated for AMD stages and thyroid autoantibody positivity.

Missing values in BMI, PIR, smoking status, hypertension, diabetes, cardiovascular disease, and free triiodothyronine (FT3) were handled using multiple imputation by chained equations. Education was complete in the analytic sample and was included as a predictor but was not imputed. Multiple imputation assumed that data were missing at random conditional on the variables included in the imputation model. Predictive mean matching was used for continuous variables and logistic regression for binary variables. The imputation model included the AMD outcome, antibody exposures, all analysis covariates, WTMEC2YR, SDMVSTRA, and SDMVPSU. Outcomes, exposures, and survey-design variables were used as predictors but were not imputed. Ten imputed datasets were generated over 10 iterations. Analyses were conducted separately within each dataset and pooled using Rubin's rules.^30^ Variable-specific missingness is reported in Supplementary Table S3.

During manuscript revision, the mutually adjusted model including both TPOAb and TgAb positivity was designated as the primary observational analysis. This designation was not prospectively prespecified. Model 1 included TPOAb and TgAb positivity simultaneously without additional covariates. Model 2 additionally adjusted for age modeled using a natural cubic spline with 4 degrees of freedom, sex, and race/ethnicity. Model 3 additionally adjusted for BMI, smoking status, PIR, education, hypertension, diabetes, and cardiovascular disease. Secondary analyses evaluated mutually exclusive antibody profiles and log2-transformed antibody concentrations. Stage-specific models compared early AMD with no AMD and late AMD with no AMD. Subgroup analyses were conducted by age (<60 vs. ≥60 years) and sex, with multiplicative interaction terms used to test effect modification. These analyses were considered exploratory, and nominal two-sided *P* values were reported without multiplicity adjustment. Sensitivity analyses included (1) complete-case analysis; (2) a nonresponse-weighted analysis in which inclusion probabilities were estimated among all 4025 age-eligible participants using age, sex, race/ethnicity, education, and PIR. Missing education was retained as a separate category; missing PIR values were replaced with the median and accompanied by a missingness indicator. Stabilized inverse-probability-of-inclusion weights were truncated at the first and 99th percentiles and multiplied by WTMEC2YR; (3) additional adjustment for thyroid-related medication use, log2-transformed thyroid-stimulating hormone (TSH), FT3, and free thyroxine (FT4); and (4) restriction to euthyroid participants with TSH of 0.34–5.60 mIU/L and FT4 of 0.60–1.60 ng/dL who were not taking thyroid-related medications.

## Results

### Characteristics of NHANES Participants

Among 4025 adults aged 40 years or older, 1058 were excluded because of missing AMD grading or thyroid antibody measurements. Compared with excluded participants, included participants were younger (weighted mean age, 56.49 vs. 59.71 years; *P* < 0.001), more likely to be non-Hispanic White, had higher educational attainment, and had a higher PIR. Sex distribution (*P* = 0.077) and BMI (*P* = 0.708) did not differ significantly between included and excluded participants (Supplementary Table S4).

Among the 2967 included participants, 234 had AMD, corresponding to an unweighted proportion of 7.9% and a weighted prevalence of 6.6% (95% CI, 5.6%–7.9%). There were 208 early AMD cases and 26 late AMD cases, with weighted prevalences of 6.0% (95% CI, 4.8%–7.4%) and 0.7% (95% CI, 0.4%–1.3%), respectively. The weighted prevalences of TPOAb positivity, TgAb positivity, and positivity for either antibody were 13.7% (95% CI, 12.1%–15.4%), 8.7% (95% CI, 7.4%–10.3%), and 17.3% (95% CI, 15.7%–19.0%), respectively. Among the 234 participants with AMD, 62 were positive for at least one antibody, including 22 with isolated TPOAb positivity, 19 with isolated TgAb positivity, and 21 with positivity for both antibodies. Participants with AMD were older and differed in race and ethnicity, poverty-income ratio, education, smoking, cardiovascular disease, thyroid-related medication use, TSH, FT3, FT4, and antibody-profile distribution compared with those without AMD (Table 1).

**Table 1.** Characteristics of Participants According to AMD Status

	All Participants (*n* = 2967)	Non-AMD (*n* = 2733)	All AMD (*n* = 234)	*P* Value
Age (y)	56.49 ± 11.44	55.76 ± 11.01	66.78 ± 12.38	<0.001
Sex				0.431
Male	1486 (48.0%)	1370 (48.2%)	116 (45.6%)	
Female	1481 (52.0%)	1363 (51.8%)	118 (54.4%)	
Race/ethnicity				0.007
Mexican American	442 (5.5%)	415 (5.6%)	27 (3.9%)	
Other Hispanic	320 (3.8%)	301 (3.9%)	19 (3.0%)	
Non-Hispanic White	1581 (77.0%)	1418 (76.4%)	163 (85.1%)	
Non-Hispanic Black	526 (8.8%)	511 (9.2%)	15 (3.0%)	
Other race	98 (4.9%)	88 (4.9%)	10 (5.0%)	
BMI	28.91 ± 6.19	28.97 ± 6.22	28.08 ± 5.67	0.100
PIR	3.33 ± 1.61	3.37 ± 1.60	2.86 ± 1.58	0.001
Education				0.042
High school or below	1621 (44.5%)	1488 (44.1%)	133 (50.9%)	
Some college or above	1346 (55.5%)	1245 (55.9%)	101 (49.1%)	
Smoking	1542 (49.9%)	1413 (49.4%)	129 (57.1%)	0.041
Hypertension	1365 (41.2%)	1236 (40.5%)	129 (51.6%)	0.112
Diabetes	453 (10.9%)	422 (10.9%)	31 (10.9%)	0.967
Cardiovascular disease	433 (11.4%)	371 (10.4%)	62 (24.7%)	<0.001
Thyroid-related medication use	260 (9.3%)	223 (8.9%)	37 (15.4%)	0.003
TSH (uIU/mL)	2.13 ± 2.41	2.11 ± 2.42	2.43 ± 2.24	0.032
FT3 (pg/mL)	3.10 ± 0.40	3.11 ± 0.40	2.97 ± 0.36	<0.001
FT4 (ng/dL)	0.78 ± 0.16	0.78 ± 0.15	0.85 ± 0.25	0.001
Thyroid autoantibody profile				0.001
Neither antibody	2471 (82.7%)	2299 (83.4%)	172 (72.9%)	
TPOAb positive only	251 (8.6%)	229 (8.6%)	22 (8.8%)	
TgAb positive only	101 (3.6%)	82 (3.2%)	19 (9.4%)	
Both antibodies positive	144 (5.1%)	123 (4.8%)	21 (8.9%)	

Data are presented as weighted means ± weighted standard deviation for continuous variables and unweighted counts with weighted percentages for categorical variables.

### Associations of Thyroid-Specific Autoantibodies With Prevalent AMD

When TPOAb and TgAb positivity were entered simultaneously, TgAb positivity was associated with higher odds of prevalent AMD in Model 1 (OR, 2.66; 95% CI, 1.58–4.49; *P* = 0.001). The association was attenuated but remained present after demographic adjustment (OR, 2.00; 95% CI, 1.13–3.55; *P* = 0.021) and in the fully adjusted model (OR, 2.02; 95% CI, 1.13–3.61; *P* = 0.022). TPOAb positivity was not associated with AMD in the fully adjusted model (OR, 0.93; 95% CI, 0.63–1.37; *P* = 0.676) (Table 2).

**Table 2.** Association of Thyroid Autoantibody Positivity and Prevalent Age-Related Macular Degeneration

		Model 1^a^		Model 2^b^		Model 3^c^	
Exposure	AMD Cases/Participants	OR (95% CI)	*P* Value	OR (95% CI)	*P* Value	OR (95% CI)	*P* Value
TPOAb positive	43 / 395	0.93 (0.62, 1.41)	0.726	0.92 (0.62, 1.34)	0.629	0.93 (0.63, 1.37)	0.676
TgAb positive	40 /245	2.66 (1.58, 4.49)	0.001	2.00 (1.13, 3.55)	0.021	2.02 (1.13, 3.61)	0.022

TPOAb and TgAb positivity were defined as antibody concentrations of ≥9 IU/mL and ≥4 IU/mL, respectively. TPOAb and TgAb positivity were entered simultaneously in all models.

a Included both antibody indicators without additional covariates.

b Additionally adjusted for age modeled using a natural cubic spline with 4 degrees of freedom, sex, and race/ethnicity.

c Additionally adjusted for BMI, smoking, poverty-income ratio, education, hypertension, diabetes, and cardiovascular disease.

In the secondary analysis of mutually exclusive antibody profiles, isolated TgAb positivity was associated with higher odds of AMD compared with negativity for both antibodies (Model 3 OR, 2.58; 95% CI, 1.42–4.69; *P* = 0.004). TPOAb positivity only and positivity for both antibodies were not statistically significant, although estimates for the double-positive group were imprecise (Supplementary Table S5). In addition, each doubling of TgAb concentration was associated with higher odds of AMD in the fully adjusted model (OR, 1.10; 95% CI, 1.01–1.21; *P* = 0.039), whereas TPOAb concentration was not associated with AMD (OR, 1.00; 95% CI, 0.95–1.06; *P* = 0.851) (Supplementary Table S6).

In stage-specific analyses, TgAb positivity was associated with early AMD (OR, 2.04; 95% CI, 1.15–3.62; *P* = 0.018) (Supplementary Table S7). The estimate for late AMD was also above 1 but was highly imprecise (OR, 2.50; 95% CI, 0.58–10.73; *P* = 0.198), reflecting the limited number of late AMD cases (n = 26). These exploratory analyses do not establish that the association differed between early and late AMD.

### Subgroup and Sensitivity Analyses of the Observational Study

No evidence of interaction by age or sex was detected (Supplementary Table S8).

The association of TgAb positivity with AMD remained directionally consistent in complete-case analysis (OR, 2.09; 95% CI, 1.07–4.08; *P* = 0.032), nonresponse-weighted analysis (OR, 2.06; 95% CI, 1.16–3.64; *P* = 0.017), and after additional adjustment for log2-transformed TSH, FT3, FT4, and thyroid-related medication use (OR, 1.96; 95% CI, 1.02–3.78; *P* = 0.044). The association was also observed among euthyroid participants not taking thyroid-related medications (OR, 3.17; 95% CI, 1.65–6.12; *P* = 0.002) (Supplementary Table S9).

### MR Results of HT and GD With AMD

In forward MR analyses, the primary IVW multiplicative random-effects estimate suggested a possible association between genetic liability to HT and AMD (OR, 1.14; 95% CI, 1.01–1.29; *P* = 0.033). The complementary fixed-effects IVW estimate was similar (OR, 1.14; 95% CI, 1.04–1.25; *P* = 0.006). No significant heterogeneity was detected for HT (Cochran's Q *P* = 0.073), and the MR-Egger intercept suggested no evidence of horizontal pleiotropy (*P* = 0.829) (Table 3; Supplementary Table S10).

**Table 3.** Mendelian Randomization Estimates for the Associations of Genetic Liability to Hashimoto Thyroiditis and Graves’ Disease With AMD

		AMD		
Exposure	Method	OR (95% CI)	*P* Value	No. of SNPs
Hashimoto thyroiditis				
	IVW (fixed effects)	1.14 (1.04, 1.25)	0.006	12
	IVW (multiplicative random effects)	1.14 (1.01, 1.29)	0.033	12
	MR-egger	1.18 (0.83, 1.68)	0.367	12
	Weighted median	1.06 (0.92, 1.22)	0.430	12
	Weighted mode	0.99 (0.63, 1.56)	0.974	12
Graves’ disease				
	IVW (fixed effects)	1.07 (1.02, 1.14)	0.012	24
	IVW (multiplicative random effects)	1.07 (1.00, 1.16)	0.060	24
	MR-Egger	1.18 (0.95, 1.46)	0.145	24
	Weighted median	1.02 (0.93, 1.11)	0.703	24
	Weighted mode	0.97 (0.75, 1.27)	0.846	24

The IVW multiplicative random-effects model was considered the primary MR estimate; fixed-effects IVW, MR-Egger, weighted median, and weighted mode were used as complementary methods.

For GD, the evidence was inconsistent. The primary IVW multiplicative random-effects estimate did not reach nominal statistical significance (OR, 1.07; 95% CI, 1.00–1.16; *P* = 0.060). Although the fixed-effects IVW estimate was nominally significant (OR, 1.07; 95% CI, 1.02–1.14; *P* = 0.012), the other complementary MR methods were not significant. Significant heterogeneity was observed for GD (Cochran's Q *P* = 0.013), although there was no evidence of horizontal pleiotropy based on the MR-Egger intercept test (*P* = 0.375).

In reverse MR analyses, there was no robust evidence that genetic liability to AMD increased the risk of either HT or GD. For HT, the IVW fixed-effects estimate was borderline significant (OR, 1.03; 95% CI, 1.00–1.06; *P* = 0.050), but the IVW multiplicative random-effects estimate was not significant (OR, 1.03; 95% CI, 0.99–1.08; *P* = 0.152). No association was observed between AMD and GD in reverse MR analyses (Supplementary Table S11). All retained genetic instruments had *F* statistics greater than 30, indicating a low risk of weak-instrument bias. Steiger directionality tests were consistent with the specified exposure-to-outcome orientation in the forward and reverse analyses (all *P* < 0.001). In leave-one-out analyses, the estimates for genetic liability to HT and GD with AMD remained directionally positive after sequential exclusion of each variant, although statistical precision varied (Supplementary Figure S1).

## Discussion

In this cross-sectional study of U.S. adults, TgAb positivity was associated with prevalent AMD after mutual adjustment for TPOAb positivity. The association was directionally consistent in mutually exclusive antibody-profile and continuous-concentration analyses and remained present across complete-case, nonresponse-weighted, thyroid-function-adjusted, and euthyroid/no-medication analyses. TPOAb positivity was not associated with AMD in the primary analysis. In MR analyses, the primary IVW multiplicative random-effects estimate suggested an association between genetic liability to HT and AMD, whereas the evidence for GD was less consistent.

These findings refine, rather than simply repeat, the existing thyroid-AMD literature. Prior studies have mainly focused on thyroid hormone status or clinically recognized thyroid disease. Higher free thyroxine concentrations were associated with increased AMD risk in the Rotterdam Study, and overt hyperthyroidism was associated with 10-year incident AMD in the Blue Mountains Eye Study.^14^^,^^15^ Nationwide studies also linked prior hyperthyroidism or hypothyroidism to prevalent AMD and thyroid dysfunction to exudative AMD.^19^^,^^20^ A meta-analysis further supported an overall positive association between thyroid disease and AMD.^16^ However, prior population-based evidence did not suggest that TPOAb was a consistently informative marker. In the Rotterdam Study, baseline TPOAb levels were nearly identical in participants who did and did not develop incident AMD.^14^ In our study, TPOAb positivity alone was not associated with AMD, whereas TgAb showed a more consistent signal across several parameterizations. Taken together, the available evidence suggests that thyroid autoimmunity may be relevant to AMD but that TgAb may have been the more informative serologic marker in the present dataset.

Stage-specific analyses did not establish that the association differed between early and late AMD. Although TgAb positivity was associated with early AMD, the estimate for late AMD was highly imprecise because only 26 late AMD cases were available. Therefore, the late-AMD findings should be considered inconclusive rather than interpreted as evidence of a weaker association at the late stage.

A second potentially relevant feature of our results is that TgAb appeared more consistently associated with AMD than TPOAb. Although TPOAb is often considered the more established serologic marker for autoimmune thyroiditis, TgAb can provide complementary information and may reflect a partly distinct dimension of thyroid autoimmunity.^31^^,^^32^ The extra-thyroidal significance of these antibodies remains incompletely understood, and our data do not support a strong mechanistic claim that TgAb is biologically more important than TPOAb in AMD. A more restrained interpretation is that TgAb captured the relevant autoimmune phenotype more effectively in this dataset.

The broader immunologic context is also noteworthy. Studies in AMD have reported enrichment of Th1- and Th17-related immune responses, with higher IFN-γ- and IL-17-associated inflammatory activity in affected individuals.^33^^,^^34^ HT likewise has a substantial Th1/Th17 component, whereas GD has a more complex and relatively more humoral pattern of immune activation.^35^^,^^36^ These overlapping immune features provide biologic plausibility for the association observed in our study and may partly explain why the exploratory MR signal was more apparent for HT than for GD. However, this interpretation should remain cautious. Thyroid autoantibodies measured in NHANES and genetic liability to HT or GD are related but nonidentical exposures, and previous MR studies have more consistently implicated genetically predicted free thyroxine in AMD than autoimmune thyroid disease broadly.^17^^,^^18^ Thus our MR findings should be viewed as suggestive and hypothesis-generating rather than confirmatory. The retained instruments were strong, Steiger testing was consistent with the specified direction, and leave-one-out estimates remained directionally consistent; however, the complementary MR methods were nonsignificant, and significant heterogeneity was observed for GD.

This study has several strengths. It used a population-based sample of U.S. adults, standardized retinal photography grading, and multiple complementary parameterizations of thyroid autoantibodies, including mutually adjusted seropositivity, mutually exclusive antibody profiles, and log2-transformed concentrations. The consistency of the findings across sensitivity analyses also supports the robustness of the main observational signal. Several limitations should also be considered. First, the NHANES analysis was cross-sectional and based on prevalent AMD, so temporality cannot be established. Second, the number of late AMD cases was limited, reducing power for stage-specific analyses. Third, thyroid autoantibodies were measured at a single time point, and NHANES did not permit direct clinical differentiation between HT and GD. Fourth, although we adjusted for major demographic and cardiometabolic factors and performed additional sensitivity analyses, residual confounding remains possible. Fifth, because 1058 age-eligible participants were excluded because of missing AMD or antibody data and included participants differed from excluded participants in several characteristics, the NHANES examination weights may not have fully corrected for item-level nonresponse. Although results were similar in a nonresponse-weighted sensitivity analysis, residual selection bias remains possible, and the final analytic sample may not fully preserve national representativeness. Finally, the MR analyses were based on predominantly European-ancestry genetic datasets and assessed genetic liability to thyroid disease rather than circulating antibody levels themselves, which limits direct comparability with the observational findings. Because FinnGen contributed to both the thyroid-disease exposure GWAS meta-analyses and the AMD outcome GWAS, some participant overlap was possible, although the exact extent could not be quantified from publicly available summary data. Participant overlap may bias two-sample MR estimates, particularly in the presence of weak instruments.^37^ Although all retained instruments had *F* statistics greater than 30, overlap-related bias cannot be entirely excluded. Accordingly, the MR findings should be interpreted as suggestive and hypothesis-generating.

## Conclusions

Thyroid autoantibodies, particularly TgAb, were associated with a higher prevalence of AMD in U.S. adults. These findings extend prior work on thyroid function and clinically recognized thyroid disease by suggesting that thyroid autoimmunity itself may also be relevant to AMD. Further prospective studies and mechanistic investigations are needed to establish temporality and determine whether thyroid autoantibodies reflect pathways relevant to AMD or broader systemic inflammatory states.

## Supplementary Material

Supplement 1

## References

[1] Klein R, Chou CF, Klein BE, Zhang X, Meuer SM, Saaddine JB. Prevalence of age-related macular degeneration in the US population. *Arch Ophthalmol*. 2011; 129: 75–80. 10.1001/archophthalmol.2010.318 21220632

[2] GBD 2019 Blindness and Vision Impairment Collaborators; Vision Loss Expert Group of the Global Burden of Disease Study. Causes of blindness and vision impairment in 2020 and trends over 30 years, and prevalence of avoidable blindness in relation to VISION 2020: the Right to Sight: an analysis for the Global Burden of Disease Study. *Lancet Glob Health*. 2021; 9(2): e144–e160. 10.1016/S2214-109X(20)30489-7 PMC7820391 33275949

[3] Wong WL, Su X, Li X, et al. Global prevalence of age-related macular degeneration and disease burden projection for 2020 and 2040: a systematic review and meta-analysis. *Lancet Glob Health*. 2014; 2(2): e106–116. 10.1016/S2214-109X(13)70145-1 25104651

[4] Smith W, Assink J, Klein R, et al. Risk factors for age-related macular degeneration: Pooled findings from three continents. *Ophthalmology*. 2001; 108: 697–704. 10.1016/s0161-6420(00)00580-7 11297486

[5] Seddon JM, Willett WC, Speizer FE, Hankinson SE. A prospective study of cigarette smoking and age-related macular degeneration in women. *JAMA*. 1996; 276: 1141–1146. 8827966

[6] Chakravarthy U, Wong TY, Fletcher A, et al. Clinical risk factors for age-related macular degeneration: a systematic review and meta-analysis. *BMC Ophthalmol*. 2010; 10: 31. 10.1186/1471-2415-10-31 PMC3009619 21144031

[7] Adamus G. Can innate and autoimmune reactivity forecast early and advance stages of age-related macular degeneration? *Autoimmun Rev*. 2017; 16: 231–236. 10.1016/j.autrev.2017.01.005 PMC5334174 28137479

[8] Morohoshi K, Goodwin AM, Ohbayashi M, Ono SJ. Autoimmunity in retinal degeneration: autoimmune retinopathy and age-related macular degeneration. *J Autoimmun*. 2009; 33(3-4): 247–254. 10.1016/j.jaut.2009.09.003 19846275

[9] Ambati J, Atkinson JP, Gelfand BD. Immunology of age-related macular degeneration. *Nat Rev Immunol*. 2013; 13: 438–451. 10.1038/nri3459 PMC3941009 23702979

[10] Antonelli A, Ferrari SM, Corrado A, Di Domenicantonio A, Fallahi P. Autoimmune thyroid disorders. *Autoimmun Rev*. 2015; 14: 174–180. 10.1016/j.autrev.2014.10.016 25461470

[11] Ragusa F, Fallahi P, Elia G, et al. Hashimotos' thyroiditis: Epidemiology, pathogenesis, clinic and therapy. *Best Pract Res Clin Endocrinol Metab*. 2019; 33(6): 101367. 10.1016/j.beem.2019.101367 31812326

[12] Yang F, Ma H, Ding XQ. Thyroid hormone signaling in retinal development, survival, and disease. *Vitam Horm*. 2018; 106: 333–349. 10.1016/bs.vh.2017.05.001 29407441

[13] Danzi S, Klein I. Thyroid disease and the cardiovascular system. *Endocrinol Metab Clin North Am*. 2014; 43: 517–528. 10.1016/j.ecl.2014.02.005 24891175

[14] Chaker L, Buitendijk GH, Dehghan A, et al. Thyroid function and age-related macular degeneration: a prospective population-based cohort study—the Rotterdam Study. *BMC Med*. 2015; 13: 94. 10.1186/s12916-015-0329-0 PMC4407352 25903050

[15] Gopinath B, Liew G, Kifley A, Mitchell P. Thyroid dysfunction and ten-year incidence of age-related macular degeneration. *Invest Ophthalmol Vis Sci*. 2016; 57: 5273–5277. 10.1167/iovs.16-19735 27716857

[16] Xu Z, Zhang M, Zhang Q, Xu T, Tao L. Thyroid Disease Is associated with higher age-related macular degeneration risk: results from a meta-analysis of epidemiologic studies. *Ophthalmic Res*. 2021; 64: 696–703. 10.1159/000515273 33596562

[17] Li X, Li H, Cheng J, et al. Causal associations of thyroid function and age-related macular degeneration: a two-sample Mendelian randomization study. *Am J Ophthalmol*. 2022; 239: 108–114. 10.1016/j.ajo.2022.01.026 35176254

[18] Ellervik C, Boulakh L, Teumer A, et al. Thyroid function, diabetes, and common age-related eye diseases: a Mendelian randomization study. *Thyroid*. 2024; 34: 1414–1423. 10.1089/thy.2024.0257 PMC11958925 39283829

[19] Hung SH, Xirasagar S, Kuang TT, et al. Association of age-related macular degeneration with prior hyperthyroidism and hypothyroidism: a case-control study. *J Pers Med*. 2022; 12: 602. 10.3390/jpm12040602 PMC9025774 35455718

[20] Boulakh L, Isaksen JL, Poulsen HE, et al. Thyroid dysfunction and exudative age-related macular degeneration—A longitudinal nationwide registry-based cohort study. *Acta Ophthalmol*. 2024; 102: 813–820. 10.1111/aos.16705 38712900

[21] Siriwardhane T, Krishna K, Ranganathan V, et al. Significance of anti-TPO as an Early Predictive Marker in Thyroid Disease. *Autoimmune Dis*. 2019; 2019: 1684074. 10.1155/2019/1684074 PMC6699358 31467701

[22] Xu L, Liu Z, Xu X, et al. Thyroid autoimmunity is associated with dietary fat consumption. *J Clin Endocrinol Metab*. 2025; 101(6): e1902–e1910. 10.1210/clinem/dgae629 39268914

[23] Liu S, Lu C, He L, et al. Vitamin E intake and prevalence rates of thyroid dysfunction and autoimmune thyroiditis: a cross-sectional analysis of NHANES Data. *Thyroid*. 2024; 34: 753–763. 10.1089/thy.2023.0561 38534308

[24] Yehuda M, Wang CH, Pak Y, Chiu KC, Gianoukakis AG. Parity and risk of thyroid autoimmunity based on the NHANES (2001-2002, 2007-2008, 2009-2010, and 2011-2012). *J Clin Endocrinol Metab*. 2017; 102: 3437–3442. 10.1210/jc.2017-00290 28911140

[25] Klein R, Davis MD, Magli YL, Segal P, Klein BE, Hubbard L. The Wisconsin age-related maculopathy grading system. *Ophthalmology*. 1991; 98: 1128–1134. 10.1016/s0161-6420(91)32186-9 1843453

[26] Klein R, Klein BE, Jensen SC, Mares-Perlman JA, Cruickshanks KJ, Palta M. Age-related maculopathy in a multiracial United States population: the National Health and Nutrition Examination Survey III. *Ophthalmology*. 1999; 106: 1056–1065. 10.1016/S0161-6420(99)90255-5 10366071

[27] Sakaue S, Kanai M, Tanigawa Y, et al. A cross-population atlas of genetic associations for 220 human phenotypes. *Nat Genet*. 2021; 53: 1415–1424. 10.1038/s41588-021-00931-x PMC12208603 34594039

[28] Kurki MI, Karjalainen J, Palta P, et al. FinnGen provides genetic insights from a well-phenotyped isolated population. *Nature*. 2023; 613(7944): 508–518. 10.1038/s41586-022-05473-8 PMC9849126 36653562

[29] Hemani G, Tilling K, Davey Smith G. Orienting the causal relationship between imprecisely measured traits using GWAS summary data. *PLoS Genet*. 2017; 13(11): e1007081. 10.1371/journal.pgen.1007081 PMC5711033 29149188

[30] van Buuren S, Groothuis-Oudshoorn K. mice: multivariate imputation by chained equations in R. *J Stat Softw*. 2011; 45(3): 1–67.

[31] Vargas-Uricoechea H, Nogueira JP, Pinzón-Fernández MV, Schwarzstein D. The usefulness of thyroid antibodies in the diagnostic approach to autoimmune thyroid disease. *Antibodies*. 2023; 12(3): 48. 10.3390/antib12030048 PMC10366745 37489370

[32] Constantinescu SM, Hospel J, Daumerie C, Alexopoulou O, Maiter D, Burlacu MC. Significance of thyroperoxidase and thyroglobulin antibodies in medically treated Graves' disease. *Eur Thyroid J*. 2023; 12(6): e230193. 10.1530/ETJ-23-0193 PMC10762548 37930957

[33] Singh A, Subhi Y, Krogh Nielsen M, et al. Systemic frequencies of T helper 1 and T helper 17 cells in patients with age-related macular degeneration: A case-control study. *Sci Rep*. 2017; 7: 605. 10.1038/s41598-017-00741-4 PMC5429667 28377586

[34] Chen J, Wang W, Li Q. Increased Th1/Th17 responses contribute to low-grade inflammation in age-related macular degeneration. *Cell Physiol Biochem*. 2017; 44: 357–367. 10.1159/000484907 29132135

[35] Wang Y, Fang S, Zhou H. Pathogenic role of Th17 cells in autoimmune thyroid disease and their underlying mechanisms. *Best Pract Res Clin Endocrinol Metab*. 2023; 37(2): 101743. 10.1016/j.beem.2023.101743 36841747

[36] Nanba T, Watanabe M, Inoue N, Iwatani Y. Increases of the Th1/Th2 cell ratio in severe Hashimoto's disease and in the proportion of Th17 cells in intractable Graves' disease. *Thyroid*. 2009; 19: 495–501. 10.1089/thy.2008.0423 19415997

[37] Burgess S, Davies NM, Thompson SG. Bias due to participant overlap in two-sample Mendelian randomization. *Genet Epidemiol*. 2016; 40: 597–608. 10.1002/gepi.21998 PMC5082560 27625185

