# Supplementary material for: Association of Thyroid Autoimmunity With Age-Related Macular Degeneration in U.S. Adults
Source: Transl Vis Sci Technol. 2026 Nov 2;15(11):3. doi: 10.1167/tvst.15.11.3 (PMC13743821; doi:10.1167/tvst.15.11.3)
Supplement: Supplementary file 1 [file tvst-15-11-3_s001.pdf]

**Supplementary Table S1. Characteristics of GWAS study included in this study.**

| Items | GWAS ID | Sample sizes | PMID | No. of variants in the source GWAS | Population | Source cohort (s) |
|---|---|---|---|---|---|---|
| GD | ebi-a-GCST90018847 | 458620 (1678 cases and 456942 controls) | 34594039 | 24189816 | European | UK Biobank and FinnGen |
| HT | ebi-a-GCST90018855 | 395640 (15654 cases and 379986 controls) | 34594039 | 24146037 | European | UK Biobank and FinnGen |
| AMD | finn-b-H7_AMD | 209122 (3763 cases and 205359 controls) | 36653562 | 16380424 | European | FinnGen |

HT: Hashimoto thyroiditis; GD: Graves' disease; AMD: Age-related macular degeneration

**Supplementary Table S2. Genetic variants used in MR analyses.**

| Direction | Exposure | SNP | Effect allele | Other allele | Beta | SE | P value | EAF | F statistic |
|---|---|---|---|---|---|---|---|---|---|
| Forward | HT | rs10494077 | A | G | 0.1198 | 0.0196 | $9.20\times10^{-10}$ | 0.2498 | 37.36 |
| Forward | HT | rs11611029 | T | C | −0.0958 | 0.0168 | $1.16\times10^{-8}$ | 0.3930 | 32.52 |
| Forward | HT | rs11889341 | T | C | 0.1245 | 0.0191 | $6.64\times10^{-11}$ | 0.2491 | 42.49 |
| Forward | HT | rs2110451 | A | G | 0.0998 | 0.0181 | $3.33\times10^{-8}$ | 0.3755 | 30.40 |
| Forward | HT | rs3184504 | C | T | −0.1790 | 0.0171 | $1.40\times10^{-25}$ | 0.6729 | 109.58 |
| Forward | HT | rs34636506 | G | A | −0.1346 | 0.0176 | $1.92\times10^{-14}$ | 0.3738 | 58.49 |
| Forward | HT | rs3997998 | T | C | −0.1646 | 0.0246 | $2.22\times10^{-11}$ | 0.1621 | 44.77 |
| Forward | HT | rs6679677 | A | C | 0.3275 | 0.0235 | $5.37\times10^{-44}$ | 0.1082 | 194.22 |
| Forward | HT | rs7030280 | T | C | 0.2051 | 0.0177 | $6.44\times10^{-31}$ | 0.7473 | 134.27 |
| Forward | HT | rs73192661 | T | C | −0.1016 | 0.0168 | $1.45\times10^{-9}$ | 0.4287 | 36.57 |
| Forward | HT | rs9271365 | G | T | 0.2531 | 0.0192 | $1.44\times10^{-39}$ | 0.4427 | 173.77 |
| Forward | HT | rs9357119 | T | G | −0.1507 | 0.0199 | $3.65\times10^{-14}$ | 0.3761 | 57.35 |
| Forward | GD | rs10821944 | T | G | −0.1566 | 0.0232 | $1.43\times10^{-11}$ | 0.7031 | 45.56 |
| Forward | GD | rs12612769 | C | A | 0.1449 | 0.0252 | $8.78\times10^{-9}$ | 0.2184 | 33.06 |
| Forward | GD | rs13136820 | T | C | −0.1361 | 0.0244 | $2.37\times10^{-8}$ | 0.7003 | 31.11 |
| Forward | GD | rs13210649 | G | T | 0.1354 | 0.0219 | $6.87\times10^{-10}$ | 0.4416 | 38.23 |
| Forward | GD | rs1569723 | A | C | 0.1641 | 0.0232 | $1.62\times10^{-12}$ | 0.7117 | 50.03 |
| Forward | GD | rs17689159 | C | T | 0.1621 | 0.0240 | $1.55\times10^{-11}$ | 0.3046 | 45.62 |
| Forward | GD | rs1977710 | G | A | 0.1461 | 0.0223 | $5.19\times10^{-11}$ | 0.4443 | 42.92 |
| Forward | GD | rs231779 | T | C | 0.2352 | 0.0222 | $3.61\times10^{-26}$ | 0.4679 | 112.25 |
| Forward | GD | rs2466028 | C | T | −0.1450 | 0.0235 | $6.28\times10^{-10}$ | 0.3610 | 38.07 |
| Forward | GD | rs28414437 | C | A | 0.2871 | 0.0228 | $1.98\times10^{-36}$ | 0.4241 | 158.56 |
| Forward | GD | rs34544259 | G | A | 0.1406 | 0.0228 | $6.75\times10^{-10}$ | 0.4047 | 38.03 |
| Forward | GD | rs4409785 | C | T | 0.2029 | 0.0350 | $6.81\times10^{-9}$ | 0.1437 | 33.61 |
| Forward | GD | rs5751536 | A | G | −0.2096 | 0.0380 | $3.37\times10^{-8}$ | 0.4511 | 30.42 |
| Forward | GD | rs61226717 | T | G | 0.2365 | 0.0357 | $3.60\times10^{-11}$ | 0.1093 | 43.89 |
| Forward | GD | rs61850863 | A | G | 0.2027 | 0.0339 | $2.18\times10^{-9}$ | 0.1450 | 35.75 |
| Forward | GD | rs6679677 | A | C | 0.3769 | 0.0536 | $2.14\times10^{-12}$ | 0.1118 | 49.45 |
| Forward | GD | rs6780858 | G | A | −0.1634 | 0.0225 | $3.85\times10^{-13}$ | 0.4681 | 52.74 |
| Forward | GD | rs6936707 | T | C | −0.4627 | 0.0479 | $4.61\times10^{-22}$ | 0.0351 | 93.31 |
| Forward | GD | rs7741597 | C | T | −0.2215 | 0.0290 | $2.27\times10^{-14}$ | 0.1441 | 58.34 |
| Forward | GD | rs7746061 | A | G | −0.3836 | 0.0516 | $1.02\times10^{-13}$ | 0.0557 | 55.27 |
| Forward | GD | rs860262 | A | C | −0.1373 | 0.0245 | $2.11\times10^{-8}$ | 0.4177 | 31.41 |
| Forward | GD | rs9260041 | T | C | −0.3419 | 0.0239 | $1.77\times10^{-46}$ | 0.2898 | 204.65 |
| Forward | GD | rs9273410 | A | C | 0.3072 | 0.0240 | $1.52\times10^{-37}$ | 0.4294 | 163.84 |
| Forward | GD | rs9357156 | C | A | 0.4396 | 0.0251 | $1.09\times10^{-68}$ | 0.2309 | 306.74 |
| Reverse | AMD | rs10754199 | G | A | −0.5774 | 0.0255 | $1.77\times10^{-113}$ | 0.5626 | 512.71 |
| Reverse | AMD | rs11200630 | C | T | 0.8323 | 0.0309 | $4.16\times10^{-160}$ | 0.2435 | 725.51 |
| Reverse | AMD | rs114003406 | T | A | 0.2876 | 0.0356 | $6.00\times10^{-16}$ | 0.1523 | 65.26 |
| Reverse | AMD | rs145630012 | G | T | −0.2945 | 0.0391 | $4.90\times10^{-14}$ | 0.1264 | 56.73 |
| Reverse | AMD | rs2230199 | C | G | 0.2138 | 0.0326 | $5.47\times10^{-11}$ | 0.1820 | 43.01 |
| Reverse | AMD | rs429608 | A | G | −0.3577 | 0.0397 | $2.14\times10^{-19}$ | 0.1200 | 81.18 |
| Reverse | AMD | rs4980260 | T | G | 0.1451 | 0.0260 | $2.42\times10^{-8}$ | 0.3776 | 31.14 |
| Reverse | AMD | rs57257925 | C | G | −0.1917 | 0.0325 | $3.63\times10^{-9}$ | 0.1891 | 34.79 |
| Reverse | AMD | rs7478014 | T | C | 0.2462 | 0.0422 | $5.51\times10^{-9}$ | 0.0998 | 34.04 |

HT: Hashimoto thyroiditis; GD: Graves' disease; AMD: Age-related macular degeneration

**Supplementary Table S3. Missingness of covariates before multiple imputation.**

| Variable | Missing, n | Missing, % |
|---|---|---|
| BMI | 21 | 0.7 |
| Smoking | 1 | 0.03 |
| PIR | 264 | 8.9 |
| Education | 0 | 0 |
| Hypertension | 2 | 0.07 |
| Diabetes | 72 | 2.4 |
| Cardiovascular disease | 21 | 0.7 |
| TSH | 0 | 0 |
| FT4 | 0 | 0 |
| FT3 | 1 | 0.03 |

BMI, body mass index; PIR, poverty-income ratio; TSH, thyroid-stimulating hormone; FT3, free triiodothyronine; FT4, free thyroxine;

**Supplementary Table S4. Characteristics of included and excluded participants aged ≥40 years in NHANES 2007–2008.**

| | Excluded (n=1058) | Included (n=2967) | P-value |
|---|---|---|---|
| Age, y | 59.71 ± 13.31 | 56.49 ± 11.44 | <0.001 |
| Sex, No. (%) | | | 0.077 |
| Male | 477 (43.3%) | 1486 (48.0) | |
| Female | 581 (56.7%) | 1481 (52.0) | |
| Race, No. (%) | | | <0.001 |
| Mexican American | 160 (6.8) | 442 (5.5) | |
| Other Hispanic | 133 (5.2) | 320 (3.8) | |
| Non-Hispanic White | 418 (61.7) | 1581 (77.0) | |
| Non-Hispanic Black | 293 (17.7) | 526 (8.8) | |
| Other race | 54 (8.6) | 98 (4.9) | |
| BMI, kg/m$^2$ | 28.81 ± 6.81 | 28.91 ± 6.19 | 0.708 |
| Education, No. (%) | | | <0.001 |
| High school or below | 712 (59.4) | 1621 (44.5) | |
| Some college or above | 342 (40.6) | 1346 (55.5) | |
| PIR | 2.61 ± 1.56 | 3.33 ± 1.61 | <0.001 |

BMI, body mass index. PIR, poverty-to-income ratio.

Data are presented as weighted means ± standard deviation or unweighted samples (weighted percentages).

**Supplementary Table S5. Associations of Mutually Exclusive Thyroid Autoantibody Profiles With AMD.**

| Exposure | AMD cases / Participants | Model 1 | | Model 2 | | Model 3 | |
|---|---|---|---|---|---|---|---|
| | | OR (95% CI) | P-value | OR (95% CI) | P-value | OR (95% CI) | P-value |
| Neither antibody | 172/ 2471 | Reference | | Reference | | Reference | |
| TPOAb only | 22 / 251 | 1.17 (0.70–1.97) | 0.519 | 1.12 (0.70–1.79) | 0.606 | 1.15 (0.71–1.85) | 0.542 |
| TgAb only | 19 / 101 | 3.37 (2.04–5.54) | <0.001 | 2.51 (1.40–4.48) | 0.004 | 2.58 (1.42–4.69) | 0.004 |
| Both antibodies | 21 /144 | 2.11 (0.96–4.64) | 0.060 | 1.57 (0.65–3.81) | 0.291 | 1.59 (0.65–3.91) | 0.289 |

AMD, age-related macular degeneration; OR, odds ratio; CI, confidence interval; TgAb, thyroglobulin antibody; TPOAb, thyroid peroxidase antibody.

The mutually exclusive antibody profile comprised four categories: neither antibody, TPOAb positivity only, TgAb positivity only, and positivity for both antibodies.

Model 1 included the antibody-profile variable without additional covariates.

Model 2 additionally adjusted for age modeled using a natural cubic spline with 4 degrees of freedom, sex, and race/ethnicity.

Model 3 additionally adjusted for BMI, smoking, poverty-income ratio, education, hypertension, diabetes, and cardiovascular disease.

**Supplementary Table S6. Associations of Log2-Transformed Thyroid Autoantibody Concentrations With AMD.**

| Exposure | Model 1 | | Model 2 | | Model 3 | |
|---|---|---|---|---|---|---|
| | OR (95% CI) | P-value | OR (95% CI) | P-value | OR (95% CI) | P-value |
| Log2-transformed TPOAb, per doubling | 0.99 (0.93, 1.05) | 0.663 | 1.00 (0.95, 1.06) | 0.951 | 1.00 (0.95, 1.06) | 0.851 |
| Log2-transformed TgAb, per doubling | 1.16 (1.06, 1.27) | 0.003 | 1.10 (1.01, 1.21) | 0.038 | 1.10 (1.01, 1.21) | 0.039 |

AMD, age-related macular degeneration; OR, odds ratio; CI, confidence interval; TgAb, thyroglobulin antibody; TPOAb, thyroid peroxidase antibody.

Log2-transformed TPOAb and TgAb concentrations were entered simultaneously in all models. ORs represent the change in the odds of AMD per doubling of antibody concentration.

Model 1 included both log2-transformed antibody concentrations without additional covariates.

Model 2 additionally adjusted for age modeled using a natural cubic spline with 4 degrees of freedom, sex, and race/ethnicity.

Model 3 additionally adjusted for BMI, smoking, poverty-income ratio, education, hypertension, diabetes, and cardiovascular disease.

**Supplementary Table S7. Associations of Thyroid Autoantibody Positivity With Early and Late AMD.**

| Exposure | Early AMD (208 cases / 2941 participants) | | Late AMD (26 cases / 2759 participants) | |
|---|---|---|---|---|
| | OR (95% CI) | P-value | OR (95% CI) | P-value |
| TPOAb positive | 0.95 (0.59, 1.50) | 0.800 | 0.51 (0.12, 2.16) | 0.339 |
| TgAb positive | 2.04 (1.15, 3.62) | 0.018 | 2.50 (0.58, 10.73) | 0.198 |

OR, odds ratio; CI, confidence interval; AMD, age-related macular degeneration

Model was adjusted for both antibody indicators, age modeled using a natural cubic spline with 4 degrees of freedom, sex, race/ethnicity, BMI, smoking, poverty-income ratio, education, hypertension, diabetes, and cardiovascular disease.

**Supplementary Table S8. Subgroup analysis.**

| Subgroup | AMD cases / Participants | TPOAb positive | | TgAb positive | |
|---|---|---|---|---|---|
| | | OR (95% CI) | P-value | OR (95% CI) | P-value |
| Age | | | | | |
| <60 | 46 / 1462 | 1.28 (0.41, 4.03) | 0.650 | 1.63 (0.34, 7.90) | 0.519 |
| ≥60 | 188 / 1505 | 0.71 (0.37, 1.35) | 0.275 | 2.25 (1.32, 3.87) | 0.006 |
| Sex | | | | | |
| Male | 116 / 1486 | 1.05 (0.46, 2.37) | 0.909 | 2.21 (0.81, 6.07) | 0.113 |
| Female | 118 / 1481 | 0.90 (0.49, 1.68) | 0.734 | 1.85 (0.87, 3.93) | 0.103 |

OR, odds ratio; CI, confidence interval

Age-stratified models were adjusted for sex and the remaining covariates. Sex-stratified models were adjusted for age modeled using a natural cubic spline with 4 degrees of freedom and the remaining covariates. The stratifying variable was omitted from the corresponding stratified model.

P for interaction:

For TPOAb positive: age 0.300, sex 0.851

For TgAb positive: age 0.584, sex 0.802

## Supplementary Table S9. Sensitivity analysis.

| | Sensitivity analysis i (n = 2598) | | Sensitivity analysis ii (n = 2967) | | Sensitivity analysis iii (n = 2967) | | Sensitivity analysis iv (n = 2543) | |
|---|---|---|---|---|---|---|---|---|
| | OR (95% CI) | P-value | OR (95% CI) | P-value | OR (95% CI) | P-value | OR (95% CI) | P-value |
| TPOAb positive | 0.93 (0.59, 1.47) | 0.750 | 0.86 (0.59, 1.25) | 0.395 | 0.96 (0.64, 1.43) | 0.827 | 0.56 (0.33, 0.95) | 0.035 |
| TgAb positive | 2.09 (1.07, 4.08) | 0.032 | 2.06 (1.16, 3.64) | 0.017 | 1.96 (1.02, 3.78) | 0.044 | 3.17 (1.65, 6.12) | 0.002 |

OR, odds ratio; CI, confidence interval; TSH, thyroid-stimulating hormone; FT3, free triiodothyronine; FT4, free thyroxine;

Stabilized inverse-probability-of-inclusion weights were estimated using age, sex, race and ethnicity, education, and poverty-income ratio, truncated at the 1st and 99th percentiles, and multiplied by the NHANES MEC examination weights.

Euthyroid status was defined as TSH of 0.34–5.60 mIU/L and FT4 of 0.60–1.60 ng/dL, with no thyroid-related medication use.

Model was adjusted for both antibody indicators, age modeled using a natural cubic spline with 4 degrees of freedom, sex, race/ethnicity, BMI, smoking, poverty-income ratio, education, hypertension, diabetes, and cardiovascular disease.

Sensitivity analysis i, complete-case analysis.

Sensitivity analysis ii: nonresponse-weighted sensitivity analysis. Missing education was retained as a separate category; missing PIR was replaced with the median and accompanied by a missingness indicator.

Sensitivity analysis iii, additional adjustment for usage of thyroid-related drugs, log2-transformed TSH, FT3, and FT4.

Sensitivity analysis iv, restricted to euthyroid participants not taking thyroid medication.

**Supplementary Table S10. Instrument Strength, Heterogeneity, Directional Horizontal Pleiotropy, and Steiger Directionality Tests**

| Exposure | Outcome | Minimum F | Cochran's *Q* test | | MR-Egger intercept test | | Correct Steiger direction | Steiger P-value |
|---|---|---|---|---|---|---|---|---|
| | | | Q | $P_{Q}$ | Intercept | $P_{intercept}$ | | |
| HT | AMD | 30.4 | 18.42 | 0.073 | -0.007 | 0.829 | Yes | <0.001 |
| GD | AMD | 30.4 | 40.70 | 0.013 | -0.021 | 0.375 | Yes | <0.001 |
| AMD | HT | 31.1 | 15.00 | 0.060 | 0.005 | 0.796 | Yes | <0.001 |
| AMD | GD | 31.1 | 7.51 | 0.483 | 0.001 | 0.966 | Yes | <0.001 |

HT: Hashimoto thyroiditis; GD: Graves' disease; AMD: Age-related macular degeneration

**Supplementary Table S11. Reverse Mendelian Randomization Estimates for the Associations of Genetic Liability to AMD With Hashimoto Thyroiditis and Graves' Disease.**

| Exposure | Methods | Hashimoto Thyroiditis | | | Graves' Disease | | |
|---|---|---|---|---|---|---|---|
| | | No. SNPs | OR (95% CI) | P-value | No. SNPs | OR (95% CI) | P-value |
| Age-related macular degeneration | IVW (fixed effects) | 9 | 1.03 (1.00, 1.06) | 0.050 | 9 | 1.01 (0.96, 1.05) | 0.780 |
| | IVW (multiplicative random effects) | 9 | 1.03 (0.99, 1.08) | 0.152 | 9 | 1.01 (0.96, 1.05) | 0.773 |
| | MR-Egger | 9 | 1.02 (0.94, 1.11) | 0.646 | 9 | 1.00 (0.92, 1.10) | 0.916 |
| | Weighted median | 9 | 1.03 (0.99, 1.07) | 0.123 | 9 | 1.00 (0.95, 1.05) | 0.906 |
| | Weighted mode | 9 | 1.02 (0.84, 1.24) | 0.815 | 9 | 1.00 (0.72, 1.39) | 1.000 |

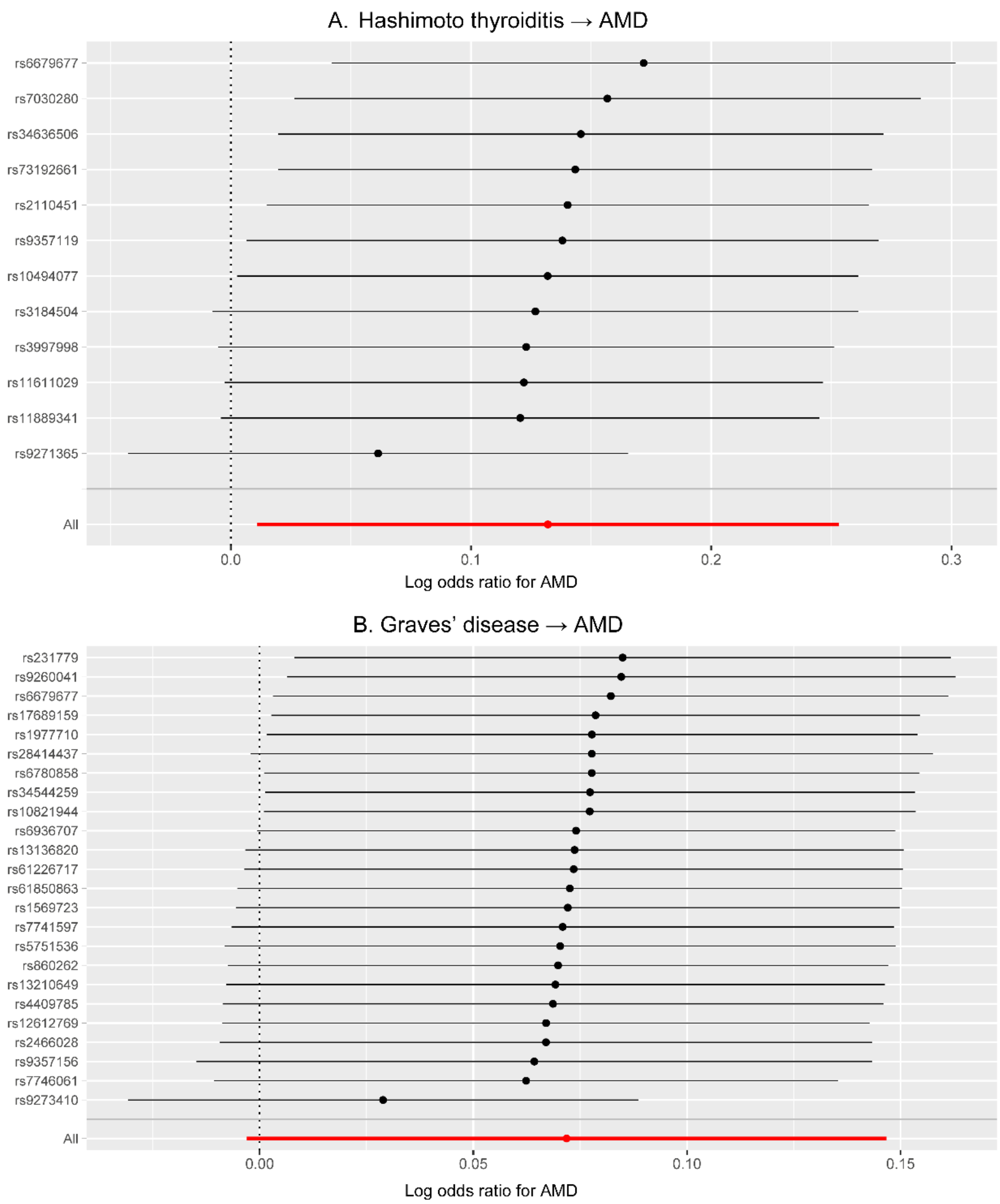


**Supplementary Figure S1. Leave-one-out analyses of genetic liability to Hashimoto thyroiditis and Graves' disease with age-related macular degeneration.** Leave-one-out analyses were performed by sequentially excluding each genetic instrument and repeating the inverse-variance-weighted analysis. Points represent effect estimates, and horizontal lines represent 95% confidence intervals. The "All" row represents the estimate obtained using all retained instruments. AMD, age-related macular degeneration; GD, Graves' disease; HT, Hashimoto thyroiditis.
